# An Advanced Multi-Analytical Approach to Study Baroque Painted Wood Sculptures from Apulia (Southern Italy)

**DOI:** 10.3390/ma19020284

**Published:** 2026-01-09

**Authors:** Daniela Fico, Giorgia Di Fusco, Maurizio Masieri, Raffaele Casciaro, Daniela Rizzo, Angela Calia

**Affiliations:** 1 Department of Engineering for Innovation, University of Salento, 73100 Lecce, Italy; daniela.fico@unisalento.it; 2 Department of Cultural Heritage, University of Salento, 73100 Lecce, Italy; giorgia.difusco@studenti.unisalento.it (G.D.F.); raffaele.casciaro@unisalento.it (R.C.); daniela.rizzo@unisalento.it (D.R.); 3 National Research Council, Institute of Cultural Heritage Sciences, 73100 Lecce, Italy; maurizio.masieri@cnr.it

**Keywords:** Italian wood sculpture, Baroque period, pigments, binders, multianalytical approach, microscopic techniques, spectroscopic/spectrometric techniques

## Abstract

Three painted valuable wood sculptures from conventual collections in Apulia (Southern Italy), made between the beginning of the 17th century and the first half of the 18th century, were studied to shed light on the pictorial materials and techniques of the Neapolitan Baroque sculpture in Southern Italy. A multi-analytical approach was implemented using integrated micro-invasive techniques, including polarized light microscopy (PLM) in ultraviolet (UV) and visible (VIS) light, scanning electron microscopy coupled with energy dispersive spectroscopy (SEM-EDS), Fourier-Transform Infrared (FTIR) spectroscopy, and pyrolysis–gas chromatography/high-resolution mass spectrometry (Py-GC/HRMS). The stratigraphic sequences were microscopically identified, and the pictorial layers were discriminated on the basis of optical features, elemental compositions, and mapping. Organic components were detected by FTIR as lipids and proteinaceous compounds for binders, while terpenic resins were detected as varnishes. Accordingly, PY-GC/HRMS identified siccative oils, animal glue, egg, and colophony. The results allowed the identification of the painting techniques used for the pictorial films and the ground preparation layers and supported the distinction between original and repainting layers. The results of this multi-analytical approach provide insights into Baroque wooden sculpture in Southern Italy and offers information to support restorers in conservation works.

## 1. Introduction

Polychromy (layers of polychrome painting) on wooden sculptures has been less investigated through analytical approaches compared to paintings on other materials, such as canvas or plasters. However, it is receiving increasing attention in analytical studies aimed at establishing the materials and techniques used for their creation, which can help art historians shed better light on this artistic production in the past [1,2,3,4], and contribute to attribution issues, to date a sculpture and to identify forgeries [5,6,7]. The study of materials and pictorial techniques used by artists in polychromy on sculpted wood surfaces may also offer fundamental data for restorers [8,9]. Indeed, a very large part of polychrome wooden sculptures consists of devotional items that have undergone frequent repainting, not only for conservation needs but also in response to changes in taste and style over the centuries, often dictated by the Church. Therefore, the study of these artifacts in many cases moves from the need to obtain evidence of past repainted layers, in order to support restoration works [10].

The potential of knowledge coming from analytical approaches is progressively involving a rich heritage of painted wood sculptures disseminated in museums and churches, as well as in convents and private collections that are often not adequately highlighted.

This is the case for Neapolitan polychrome wooden sculpture of the Baroque period (17th–18th centuries) in Southern Italy. Knowledge of this production has made significant progress in recent decades, but still remains incomplete [11,12]. On the one hand, a vast number of sculptures have emerged in various places of Southern Italy, yet many areas still remain unexplored. On the other hand, recently rediscovered sculptures have been analyzed mainly through stylistic and iconographic approaches, with less attention focused on material aspects and execution techniques [13,14]. The poor knowledge of the materials and artistic techniques used in Neapolitan sculpture in the late 17th and 18th centuries makes it difficult to clearly delineate the artistic personalities active in this period or to understand the intense political and cultural exchanges between Spain and the Viceroyalty of Naples, a period characterized by important artistic exports to Apulia in Southern Italy. Indeed, only a very few publications offer adequate technical investigations [12,15]. Some sculptures by Gaetano Patalano, one of the most important Italian sculptors of the late 17th century [16], are among the best investigated. Non-invasive and micro-invasive techniques have highlighted material peculiarities as a signature of this artist compared to most of his contemporary colleagues [17,18,19], confirming once again that analytical studies combined with art-historical and documentary studies can provide a considerable added value of knowledge, helping to resolve issues of attribution and conservation and restoration.

In this paper, three painted Baroque wooden sculptures from conventual collections in Apulia (Southern Italy) were investigated to provide insights into the painting materials and techniques used by the artists and to differentiate between original and non-original polychromies. To investigate sub-surface layers and identify original ones, micro-samples were taken and a laboratory-based analytical approach was adopted.

Today, many analytical techniques have been developed for the study of cultural heritage, and a wide range of advanced non-destructive or micro-invasive technologies applied to the study of works of art [20,21,22] are able to answer art-historical questions and support conservation and restoration interventions. Nonetheless, laboratory analyses on samples remain necessary to gain certain knowledge, such as sub-surface sequence layers, organic binders, and the quantitative composition of constituent materials. In this case, the sustainability of analytical investigations, in relation to the fundamental need to preserve the integrity of artifacts as much as possible, is addressed in terms of micro-invasive analytical protocols, and a multi-analytical approach allows for the optimization of material knowledge through the application of crossed techniques, in the context of low-impact sampling.

In the present study, microscopic techniques such as polarized light microscopy (PLM) in ultraviolet (UV) and visible (VIS) light, along with scanning electron microscopy equipped with EDS (SEM-EDS), were used to identify stratigraphy, elemental composition, and mapping of the pictorial layers. Spectroscopic and spectrometric techniques, such as micro-invasive Fourier-Transform Infrared (ATR-FTIR) spectroscopy and advanced pyrolysis–gas chromatography/high-resolution accurate mass spectrometry (Py-GC/HRMS), were implemented to detect organic binders and varnishes.

Among laboratory techniques, PLM and SEM are widely used in the study of paintings, as they provide basic information on layer sequences, morphology, structure, and the nature of some pigments [7,23,24]. Furthermore, coupling SEM with energy dispersive X-ray spectroscopy allows identification of the elemental composition of pigments and their distribution within the polychromy [24]. Combination with spectroscopic and spectrometric techniques further enriches information on painting materials and artistic techniques through the identification of organic components.

As is well-known, the ATR-FTIR technique combines infrared spectroscopy with attenuated total reflection, providing chemical information on solid and complex samples without compromising the integrity of the sample [25,26]. It is valuable for sensitive surfaces that do not need to be destroyed, both for preventive conservation and for historical and artistic research [7,23]. Moreover, advanced instrument configurations equipped with an array detector and an optical microscope allow the creation of detailed chemical maps of organic and inorganic materials, providing information on the distribution and composition of materials within a polychrome artwork [23,25,26,27].

Analytical pyrolysis (Py), especially when coupled with gas chromatography and high-resolution mass spectrometry (Py-GC/HRMS), is a powerful and versatile analytical technique for the study of complex organic materials such as paints, resins, binding media, lignocellulosic materials, fibers, and other materials of historical and artistic interest [28,29,30,31]. It is a micro-destructive technique capable of providing detailed information on the origin and structure of these materials’ components while using a minimal sample amount (50–100 mg) [32,33].

## 2. Materials and Methods

### 2.1. Materials

**Figure 1:**
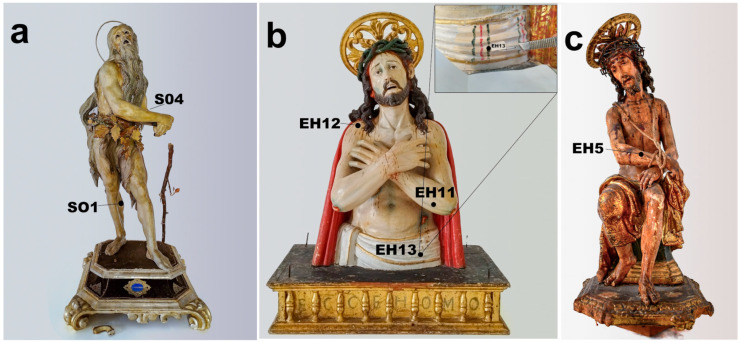
Polychrome wooden sculptures of (**a**) *St. Onofrio*, (**b**) *Ecce Homo* 1, and (**c**) *Ecce Homo 2*, before restoration works (photography taken under controlled studio conditions: tripod-mounted camera, artificial daylight-balanced softbox illumination (5500 K); no optical magnification, standard photographic reproduction ratio) with indications of the samples taken.

The statues of St. Onofrio and Ecce Homo (hereinafter referred to as Ecce Homo 1), from the collection of the Benedictine Order of Lecce (Italy), and the statue of the Ecce Homo (hereinafter referred to as Ecce Homo 2) belonging to the artistic collection of the Clarisse Order of Nardò (Lecce, Italy (Figure 1)), were investigated in this study. The historical information about the sculptors and their artistic and social context has been fully investigated in [11].

#### 2.1.1. St. Onofrio Statue

St. Onofrio (Figure 1a) is a statue 59 cm high, attributed to the famous sculptor Giacomo Colombo (Este, Padua, 1663–Naples, 1731) in the early 18th century. The definite attribution derives not only from the recovery of archival documents but also from the excellent execution quality, peculiar to Colombo, who was a painter and a sculptor. Computerized Axial Tomography (CAT) analysis highlighted the technique of assembling wooden dowels, and xylotomic analyses revealed that the statue was made of linden wood [11].

Despite its small format, the polychrome statue of St. Onofrio shows a refined anatomical rendering, leading back to probable live nude studies conducted by the artist, which is evident in the treatment of the musculature, the details of the calves, knees, arms, and the face rich in typical Baroque pathos. The sculpture appeared heavily repainted and manipulated.

#### 2.1.2. Ecce Homo 1 Statue

Ecce Homo (Figure 1b) is an all-round sculpture about one meter high, representing the bust of the wounded and bleeding Jesus. The sculpture was created in 1674 and, thanks to a previous CAT study that revealed a cartouche containing the artist’s signature concealed inside the sculpture, it has been attributed to the Spanish sculptor Diego Vigliavolos [11]. The same analysis also revealed that the sculpture was made from a single wooden block, except for the base and skull, while xylotomic analyses revealed that elm wood, a species probably imported to Apulia and not usually used in statuary, was employed. The face of Ecce Homo is crowned with a double branch of spines and framed by dense, elaborately carved hair. The sculpture exhibited heavy repainting, and the base had a black synthetic repainting with several lacunas.

#### 2.1.3. Ecce Homo 2 Statue

Ecce Homo (Figure 1c) is approximately 45 cm high and is characterized by refined polychromy and anatomical detail. The precious polychrome sculpture was probably made for private devotion in the early decades of the 17th century by the Italian sculptor Antonio Gallo and consists of a single block of cherry wood, except for the square base [11,17]. The sculptor’s attention to naturalistic and decorative aspects reveals great skill, evident especially in the anatomical details of the face and in the fabric draping the waist, which is entirely decorated in *estofado*, with polylobate panels featuring phytomorphic motifs, enclosed along the edge by a repeated motif of volutes with whirls and leaves. Pictorial retouches and lacunas were present in some areas of the surface, as well as missing or replaced elements (such as fingers of the hand substituted with terracotta).

### 2.2. Methods

Sampling (Figure 1) was performed on the occasion of the restoration and focused on the flesh portions. The choice of sampling spots was made according to these concepts: avoiding areas with small painted details (eyes, mouth, nails), which would be mutilated by the removal of even very small sections; avoiding areas that were apparently scratched; choosing areas where a high level of overpainting was visible and where the chance of identifying the original polychromy levels was more promising. Only in one case (sample EH 13) did sampling involve the loincloth of the Ecce Homo 1 statue, specifically to ascertain the originality of the decorative pattern with green and red bands, which was glimpsed under a white repainting level (detail in Figure 1b).

The following analyses were performed on sample fragments and their polished cross-sections.

- Observations by PLM

After microscopic observation of the sample fragments in their original state, polished cross-sections were prepared by embedding the pictorial samples in a polyester resin (Colorchimica SRL, Reggello, Italy). Then, the embedded samples were carefully polished using silicon carbide abrasive papers of progressively finer FEPA-standard grain sizes (P800, P1200, and P2400) by means of a grinding and polishing machine (Saphir 550, ATM GmbH, Mammelzen, Germany). During the grinding and polishing operations, the sections were thoroughly washed with ultrapure water to remove any abrasive residue.

The microscopic study was carried out by means of a polarized light optical microscope (model Eclypse 100N LV POL, Nikon Europe B.V, Amstelveen, The Netherlands), using visible and UV light in the reflection mode. Photographic images were recorded using a Nikon DS-Ri1 camera (Nikon Europe B.V, Amstelveen, The Netherlands). Microscopic observations focused on the identification of stratigraphic sequences, the features of each pictorial layer, and optically recognizable components.

- SEM-EDS analyses

Cross-sections of the pictorial layers were analyzed in low-pressure mode (90 Pa), at 25 KeV or 30 KeV (depending on the samples), without conductive coating. A scanning electron microscope (model EVO 15, by Zeiss, Jena, Germany), equipped with a tungsten filament and operating at 30 keV with a resolution of 3.4 nm, was used. EDX spectra and distribution maps of the elements were acquired under the following operational parameters: image scan size of 512 pixels along x; energy range of 10KeV; 1024 channels; spectra acquisition time of 20 s; and, for distribution maps, a frame count of 5, pixel dwell time of 1ms, and process time of 4. EDX analyses were performed using the AZtec 6.0 SP1 software platform version and an UltimMax 40 mm2 SDD detector (Oxford Instruments, Abingdon, UK), with a resolution of 127 eV FWHM @MnK and a detection limit of about 0.1 wt% percentage from 0.3 to 3 µm in depth. The software module uses a standardless ZAF quantification system (AutoPhaseMap mode). SEM-EDS analysis was aimed at investigating the morphological–structural properties of the pictorial layers and performing qualitative and semi-quantitative elemental characterization, identifying the distribution of elements in the samples.

- µATR-FTIR analyses

Molecular chemical characterization was performed on the sample fragments as collected (for top and bottom layers) or their cross-sections with a Lumos II FT-IR micro-spectrophotometer (Bruker Optics GmbH, Ettlingen, Germany) equipped with a microscope, a Germanium Attenuate Total Reflection (ATR) crystal, and an integrated room temperature MCT detector. Analyses were conducted over a scan range of 4000 cm^−1^ to 650 cm^−1^, with a resolution of 4 cm^−1^ and scan numbers varying from 64 to 200. The acquired µATR-FTIR spectra were processed with OPUS-IR™ 7.5 software version (Bruker Optik GmbH, Ettlingen, Germany), and their interpretation was carried out by comparison with reference databases and scientific literature. Five measurements were conducted on each sample for statistical treatment of the data. µATR-FTIR analyses were focused on identifying the main organic and inorganic components present in the samples. Where possible, the stratigraphic distribution of these components was also addressed using the microscope. In some samples, it was not possible to locate components due to the small thicknesses of some pictorial layers relative to the instrumental resolution.

- PY-GC/HRMS analyses

Pyrolysis–gas chromatography/high-resolution accurate mass spectrometry analysis was performed on sample paint fragments (about 0.1 mg), which were manually homogenized with 5 μL of tetramethylammonium hydroxide (TMAH, 2.5% in MeOH) to assist the hydrolysis and methylation of compounds and, after solvent evaporation at 60 °C, placed in ferromagnetic tubes having a Curie point temperature of 500 °C. The pyrolysis time was 10 s. The interface temperature of the pyrolyzer was 300 °C, and the temperature of the GC injector was kept at 280 °C. The pyrolysates were separated on a Thermo Scientific™ TraceGOLD™ TG-5SilMS 30 m × 0.25 mm I.D. × 0.25 µm film capillary column (P/N 26096-1420) using the following GC program: 40 °C (2 min), 10 °C/min to 320 °C (10 min). A Frontier Lab Multi-Shot Pyrolyzer™ model EGA/PY-3030D (Frontier Lab, Fukushima, Japan), coupled to a Thermo Scientific™ TRACE™ 1310 gas chromatographic system (Thermo Fisher Scientific, Bremen, Germany) and a Thermo Scientific™ Exactive™ GC Orbitrap™ mass spectrometer (Thermo Fisher Scientific, Bremen, Germany), was used. Compound identification was carried out by computer matching of the resulting mass spectra with the NIST mass spectral library or by comparison with mass spectra of well-known standards in the literature.

## 3. Results

### 3.1. St. Onofrio Statue

#### 3.1.1. SO1 Sample

**Figure 2:**
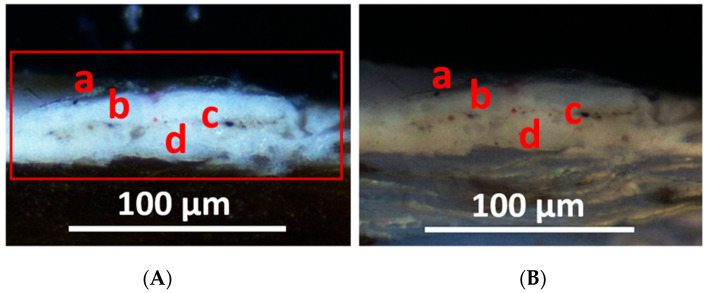
SO1 polished cross-section sample showing the sequence of layers (a–d) on the wood support: (**A**) VIS light; (**B**) UV light evidencing straw-yellow fluorescence of layer (d).

Visible light microscopic observations reveal the following stratigraphy (Figure 2):

(a) A layer consisting of a discontinuous surface varnish.
(b) A pictorial layer that appears homogeneous, with a thickness varying approximately between 15 and 20 μm. Under visible light, it is white and contains sporadic red particles measuring between about 2 and 20 μm.
(c) A very thin and discontinuous dirt deposition layer on a previously exposed surface.
(d) A layer with a thickness of about 20 μm; it is white in visible light, while it shows diffuse straw-yellow fluorescence under UV light and contains very few fine red particles with sizes from a few microns up to 15–20 μm.

**Figure 3:**
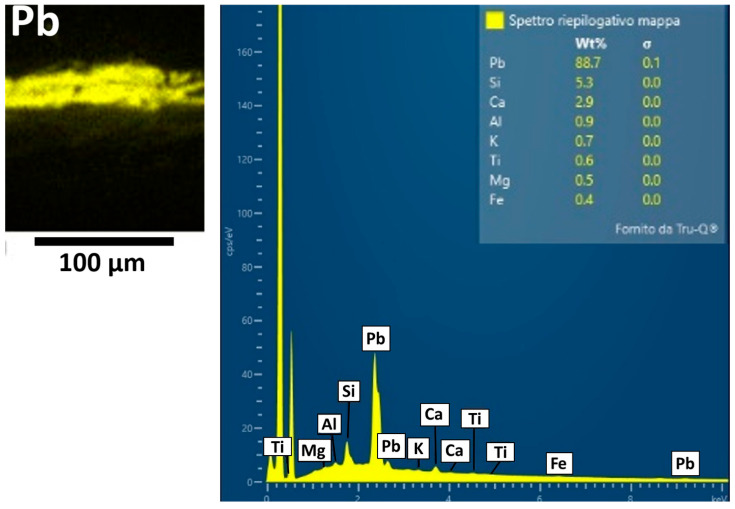
SEM-EDS spectrum of the elemental composition and Pb distribution map of SO1 sample.

SEM-EDS analysis shows the presence mainly of Pb in layers (b) and (d) (Figure 3), which suggests the use of white lead as the pigment. Traces of Si, Al, Mg, Ca, K, Fe, and Ti were also detected and are likely related to aluminosilicates constituting ochre, corresponding to the red pigment sporadically observed under PLM. Ca, Al, Si, K, Mg, Fe, and Na were detected in the earthy deposit layer (c).

ATR-FTIR analysis on the sample surface yields a band at 3400 cm^−1^ due to the stretching vibration of hydroxyl groups (–OH) and acute peaks at 2964, 2936, and 2861 cm^−1^, due to the stretching (asymmetric and symmetric) of methylene and methyl groups (–CH). A carbonyl band is characterized by a doublet from ester (1738 cm^−1^) and acid (1710 cm^−1^) groups. In the fingerprint region, there are also some infrared bands at 1314 cm^−1^ (C–H deformation), 1228, 1174, and 1074 cm^−1^ (C–O vibrations), and 985 cm^−1^ (aromatic compounds). These peaks may indicate the presence of a resin-based varnish and a lipidic binder in the pictorial layer [12]. ATR-FTIR analysis also reveals bands at 1402 cm^−1^ and 1528 cm^−1^. The former is probably attributable to basic lead carbonate (PbCO_3_)_2_·Pb(OH)_2,_ identified as the pigment in pictorial layer (b) according to SEM-EDS results. The peak at 1528 cm^−1^ comes from lead carboxylates [26], suggesting the use of an oil binder, as these compounds may derive from the interaction of a lipidic component with basic lead carbonate [34,35].

**Figure 4:**
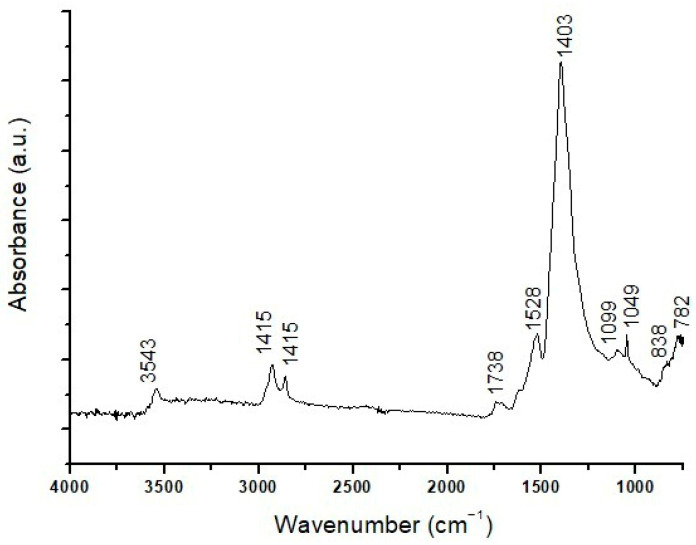
ATR-FTIR spectrum of layers (b–d) in the SO1 sample.

ATR-FTIR analysis of layers (b–d) (Figure 4) confirms the presence of basic lead carbonate as a pigment through characteristic infrared bands at 3543 cm^−1^ (stretching vibration of the O–H group), 1403 cm^−1^ (vibrations of C–O groups typical of carbonates), 1099 and 1049 cm^−1^ (C–O stretching vibrations of the CO_3_^2−^ carbonate group), 838 and 782 cm^−1^ (associated with Pb–O and C–O bond vibrations), and 682 cm^−1^ (deformation vibration of the carbonate group). Signals of the aluminosilicates related to red earth are not uniquely detectable, as they could be covered by those of the lead carbonate, which is present in notably higher amounts. There are peaks at 1169 and 1033 cm^−1^ in the spectrum, which could be attributable to both pigments and their C–O bond elongation vibrations; however, the characteristic red earth infrared band at 908 cm^−1^, typically associated with Fe–O vibrations (iron oxides), is absent. Weak infrared bands are also observed at 2962, 2938, and 2858 cm^−1^, related to CH_2_ aliphatic chains, and at 1736 cm^−1^, related to the carbonyl group (C=O), all indicative of the presence of a lipid medium. Finally, an intense peak at 1523 cm^−1^ is indicative of lead carboxylates, coming from the interaction between basic lead carbonate pigment and the siccative oil medium, as previously commented.

Pyrolysis of the overall layers (Table 1 and Table 2) shows the presence of mono- and dicarboxylic fatty acids. The high proportion of dicarboxylic acids (ΣD)—mainly azelaic, suberic, and sebacic acids—and an A/P (azelaic/palmitic acid) ratio >1 support the use of a siccative oil [36,37]. In the same pyrogram, abietanes indicative of a terpenic resin are present: dehydroabietic and 7-oxo-dehydroabietic acids suggest that the varnish on the surface is based on colophony plant resin [36,38]. Py-GC/HRMS analyses of layers (b–d) indicate the presence of mono- and dicarboxylic acids. The high content of fatty acids (FAs), dicarboxylic acids (ΣD), azelaic acid, and the A/P ratio value confirm the use of a siccative oil as the pictorial binder.

#### 3.1.2. SO4 Sample

**Figure 5:**
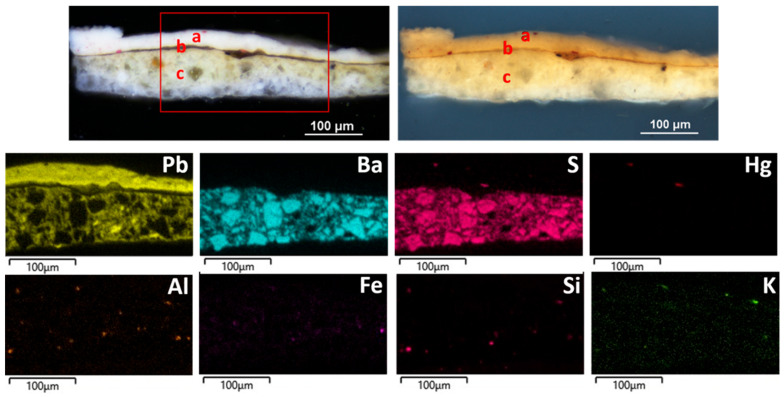
SO4 sample stratigraphy observed on polished cross-section under PLM with VIS light (**top left**) and UV light (**top right**), along with SEM-EDS maps of the elements detected in the red-outlined area (**on the bottom**).

Microscopic observation reveals the following three layers (Figure 5), moving from the surface:

(a) A layer, white in VIS light, is pigmented by diffuse red particles, some of them coarse (up to about 20 μm). Under UV light, it shows a yellowish color. The thickness ranges from about 25 to 55 μm, and the structure is fine and uniform. The features of this layer appear quite similar to those of layer d) in direct contact with the wooden substrate observed in the SO1 sample.
(b) A layer, consisting of a brown varnish, approximately 8 to 15 μm in thickness.
(c) A layer, in VIS light, it is white at the bottom and yellowish at the top, across a thickness from 10 μm to about 60 μm. This tone could be due to the presence of very fine orange-red particles, 2–3 μm in size, which are absent in the lower part of the layer. It could also result from impregnation by an organic component, such as the varnish from the overlying layer (b). This hypothesis is supported by the fact that no separation is visible between these two parts of the layer and, in addition, a yellow color is also evidenced under UV light in the upper part of layer (c). Throughout the overall layer, having a thickness between 60 and 85 μm, the structure is made of a fine matrix containing translucent, sharp-edged crystals with coarse sizes, up to about 30 μm.

SEM-EDS spectra and distribution maps of the elements (Figure 5) allow the identification of white lead as the main pigment in layer (a) and coarse crystals of barium white, mixed with white lead, in layer (c). In both layers, traces of elements related to aluminosilicate in red earth pigments are present; traces of Hg in layer (a) denote the use of cinnabar.

Overall, the ATR-FTIR analyses was performed on the SO4 sample as it was revealed the presence of a resin, with characteristic peaks at 3400, 2932, 2865, 1716, 1266, 1169, 1119, 1072, and 989 cm^−1^. As pigments, lead white was identified, with typical bands at 3554, 1406, 825, and 686 cm^−1^, and red earth, with peaks at 1622 cm^−1^ (O–H group bending vibration), 1058 cm^−1^ (stretching vibration of the C–O group), and 933 and 778 cm^−1^ (C–H and O–H vibrations of clay groups).

As in the SO1 sample, a lipidic component was detected, with signals at 2926, 2858, and 1737 cm^−1^, as well as a band at 1536 cm^−1^, attributable to carboxylates.

**Figure 6:**
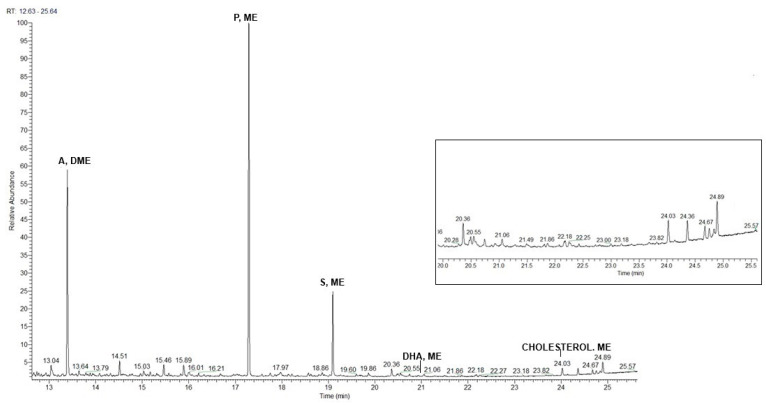
Pyrogram of (a) and (b) layers in the SO4 sample.

Pyrolysis of the SO4 sample, including (a) and (b) layers (Figure 6), shows the presence of mono- and dicarboxylic acids, protein compounds, and abietans (Table 1 and Table 2). The fatty acid content, the ratios of palmitic/stearic acids and azelaic/palmitic acids, the azelaic acid content <10%, and the presence of markers such as cholesterol, derivatives, and hexadecanenitrile identify a mixture of oil and egg, suggesting the use of a so-called *tempera grassa* in the (a) pictorial layer [12,36]. Retene, dehydroabietic, and 7-oxo-dehydroabietic acids are also present in the pyrogram and led to the identification of colophony, subjected to heating and aging processes [36,38], as the varnish used in the (b) layer.

High percentages of mono- and dicarboxylic fatty acids are present in the pyrogram, including the (b) and (c) layers. The A/P ratio >1 and the azelaic acid content of 34.95% indicate the use of a siccative oil as a binder in the (c) painted layer [36,37]. Traces of abietanes are also present in the pyrogram, relating to the colophony in the (b) layer.

### 3.2. Ecce Homo 1 Statue

#### 3.2.1. EH11 Sample

**Figure 7:**
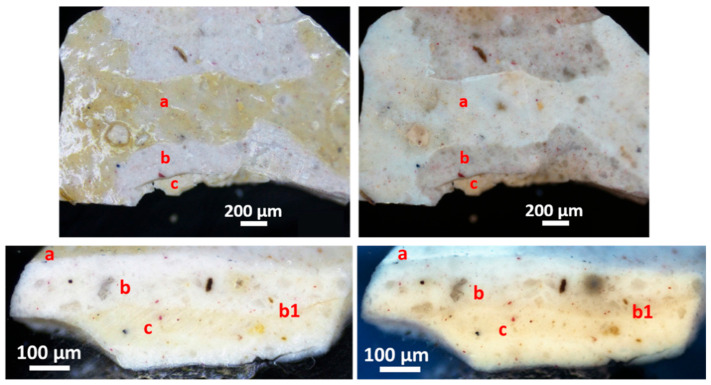
EH11 sample fragment as it is (**top**) and in polished cross-section (**bottom**) under VIS and UV light (**left** and **right**, respectively).

The sample fragment shows the presence of three layers, which are clearly visible under UV light on the sample as it is. In particular, the following layers (Figure 7):

(a) The surface layer shows a white fluorescence and consists of a varnish.
(b) The pictorial layer is grayish white.
(c) The white-pale yellow pictorial layer has a pinkish fluorescence.

In polished cross-section under visible light (Figure 7), layers (b) and (c) appear to consist of a white pigment containing black and red particles; they have yellowish color under UV light and coarse thicknesses of about 110 and 150 μm, respectively. The two layers differ in the dimensions of the black pigment and in their structure. The black pigment is coarse in the (b) layer (from 10 μm up to 35 μm), whereas it is sparser and finer (between 4 μm and 11 μm) in the (c) layer. In addition, the structure of layer (b) is characterized by a fine white pigment matrix containing a few translucent, colorless, sharp-edged crystals with dimensions from approximately 15 to 55 μm, while the white pigment in layer (c) is uniformly fine-grained. Finally, the stratigraphic section reveals the presence of a very thin layer (b1) between layers (b) and (c), with the characteristics of a colorless varnish.

In both layers (b) and (c), SEM-EDS analyses detected Pb, relating to white lead as the main pigment. The elemental composition (Si, Al, Mg, Fe, and Hg) suggests red ochre, cinnabar, and black carbon as secondary pigments added to the white lead.

**Figure 8:**
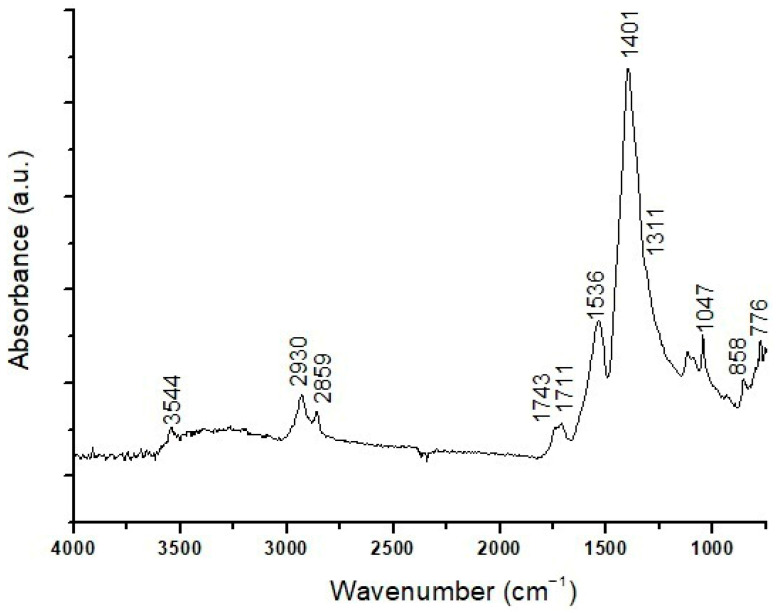
ATR-FTIR spectrum of the EH11 sample.

The µATR-FTIR analyses performed on the surface of the sample (Figure 8) identified a resin, with characteristic peaks at 3400, 2930, 2859, 1711, 1311, 1047, and 931 cm^−1^, and a lipidic component related to the bands at 2930, 2859, 1743, 1121, and 1086 cm^−1^. Peaks at 3544, 1401, 858, and 776 cm^−1^ indicate the presence of lead carbonate, and a peak at 1536 cm^−1^ can be associated with lead carboxylates. Below the surface, the composition of each layer observed microscopically was not discriminated; the overall composition detected lead carbonate, with characteristic infrared peaks at 3546, 1745, 1626, 1398, 1103, 1050, 836, and 776 cm^−1^. No infrared bands correlated with the red pigment were observed, probably due to the coverage by the bands of white lead present in higher amounts.

**Figure 9:**
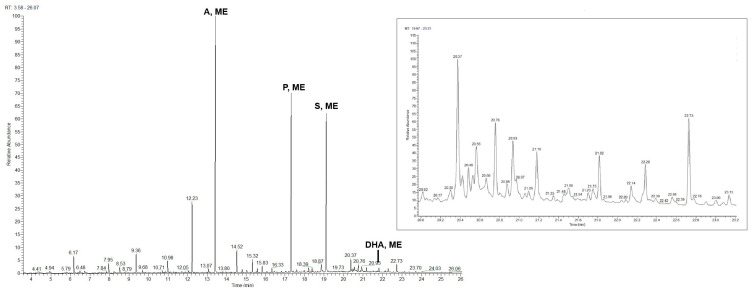
Pyrogram of the EH11 sample.

Py-GC/HRMS analyses of the overall EH11 sample (Figure 9) indicate the presence of mono- and dicarboxylic acids. The high content of fatty acids, dicarboxylic acids, azelaic acid, and the A/P ratio value indicate the use of a siccative oil as the pictorial binder. Specifically, based on the P/S ratio usually used in the literature to identify the nature of oils [36,37,39], it is possible to assume the use of linseed oil. Retene, dehydroabietic, 7-methoxy-tetradehydroabietic, and 7-oxo-dehydroabietic acids, also present in the pyrogram, indicate the use of colophony as a varnish and that it was subjected to heating and aging processes, also in this case [36,38].

#### 3.2.2. EH12 Sample

**Figure 10:**
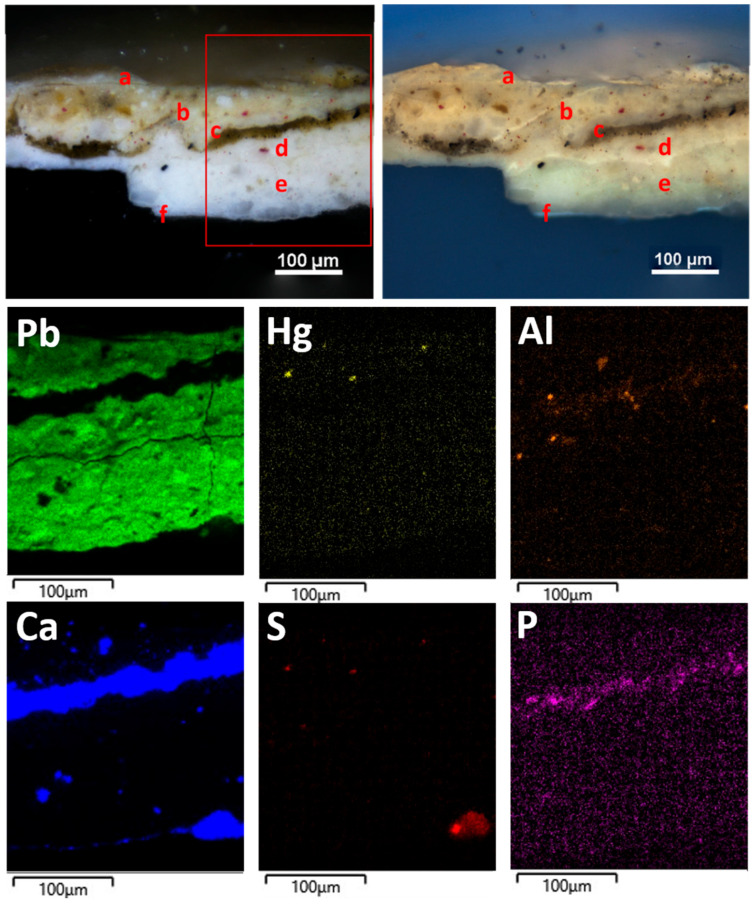
EH12 sample: micrographs of the stratigraphic sequence on cross-section under VIS and UV light (**top left** and **right**, respectively), along with SEM-EDS maps of the elements detected in the red-outlined area.

The sample EH12 consists of five layers. Moving from the top, the following were microscopically observed (Figure 10):

(a) A layer of varnish.
(b) A layer about 90 μm thick, white in color, showing diffuse small red particles (about 6–8 μm in size) and sporadic smaller black particles.
(c) A layer approximately 20 μm thick, consisting of a very intense black pigment and diffuse carbonaceous particles with a maximum dimension of 14 μm; in some spots of the sample as it is, a red pigment is visible in addition to the black one.
(d) A layer about 75 μm thick, white in color, containing orange-red particles (maximum sizes between 6 and 14 μm) and sharp-edged translucent crystals with dimensions from about 15 to 45 μm.
(e) A layer about 70 μm thick, made of a fine white pigment and including translucent, sharp-edged, coarse crystals ranging from about 10 μm to 50 μm. It contains sporadic fine yellow–red particles (about 5–7 μm size) and more diffuse, coarser black particles with sizes of about 10–13 μm.
(f) A very thin layer (less than 10 μm in thickness), white under VIS light and showing a bluish UV fluorescence.

It should be noted that the microscopic features of layer (e) appear to be very similar to those observed for the (b) layer of the EH11 sample. On the other hand, the (c) layer probably relates to color smearing during the creation of artistic details, such as blood/hair, close to the sampling point, which was then covered by the white painting ((b) layer).

The elemental composition determined by SEM-EDS analyses (Figure 10) shows the presence of Pb in layers (b), (d), and (e), indicating white lead as the white pigment. Traces of Hg in layer (b) and Al detected in layers (b) and (d) suggest cinnabar and ochres as red pigments added to the white lead.

**Figure 11:**
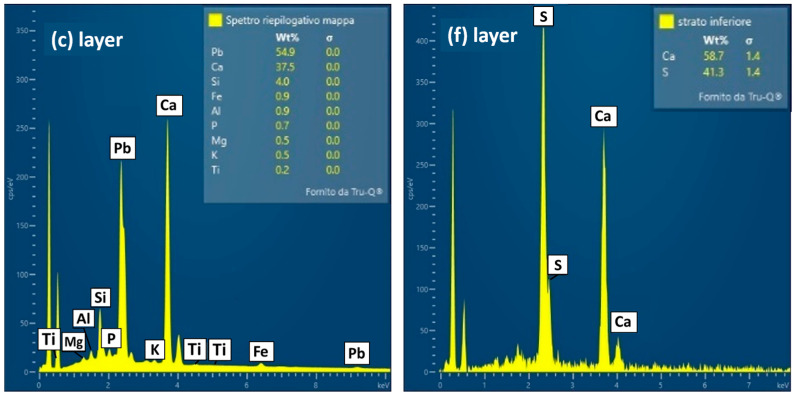
SEM-EDS spectra of layers (c) and (f) within the EH 12 sample.

The (c) layer (Figure 11) has a composition mainly made of calcium, suggesting calcium carbonate as the pigment, and a secondary presence of elements (Al, Si, Mg, Fe) in the SEM-EDS spectrum, probably relating to the addition of red ochre. Indeed, in some spots of the sample as it is, a red pigment is visible in addition to the black one. P is also present and accounts for the black pigment consisting of bone black.

The composition of the ground layer (f) (Figure 11) is made of Ca and S, suggesting that it is most likely an original gypsum-based preparatory layer supporting the application of the pictorial film.

µATR-FTIR analyses of the part of the sample from the surface up to layer (c) reveal the presence of intense bands at 3685, 1622, 1100, 1044, and 777 cm^−1^, which can be attributed to aluminosilicates coming from ochres. In addition, infrared peaks related to lead carbonate (3545, 1402, 1047, and 841 cm^−1^), a lipid component (2937, 2864, and 1725 cm^−1^), and carboxylates (1545 cm^−1^) appear in the ATR-FTIR spectrum.

**Figure 12:**
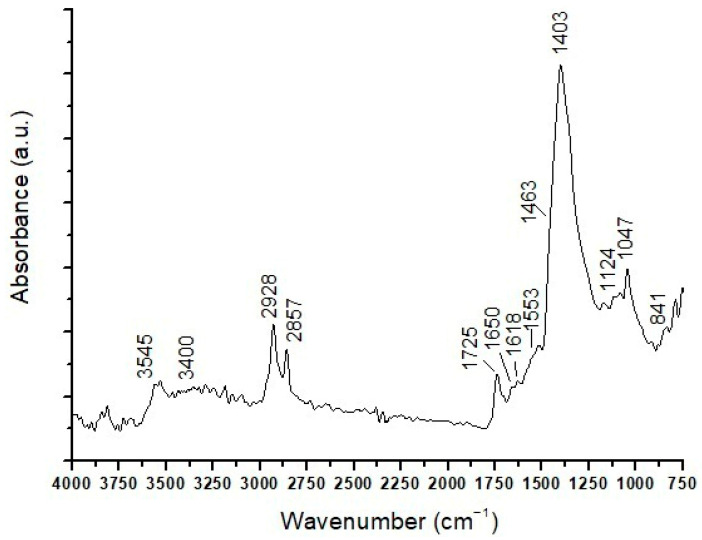
ATR-FTIR spectrum of layers (d), (e), and (f) in the sample EH12.

Similarly, in the lower area of the sample, comprising layers (d), (e), and (f) (Figure 12), there are bands attributable to lead carbonate, a lipid component, and aluminosilicates. Moreover, infrared bands at 3400 cm^−1^ (N–H stretching band), 2928 and 2857 cm^−1^ (hydrocarbon stretching), and 1650, 1553, and 1463 cm^−1^ (attributed to amide I, amide II, and amide III, respectively) of proteinaceous compounds [26,37] appear in the ATR-FTIR spectrum of the lower areas of the sample. The presence of proteins supports the hypothesis that layer (e), which fluoresces blue under UV light, constitutes a proteinaceous preparation layer applied to the wood substrate prior to painting.

PY-GC/HRMS analysis of layers (a), (b), and (c) shows a considerable abietane content (22.74%), indicative of a terpenic resin likely used as a varnish on the surface. Specifically, retene, dehydroabietic acid, 7-methoxy tetrahydroabietic acid, and 7-oxo-dehydroabietic acid are attributable to colophony subjected to oxidation processes, as well as heating at temperatures above 300 °C. Mono- and dicarboxylic acids are also present in the pyrogram. The A/P ratio, the low azelaic acid content, and the presence of hexadecanenitrile [36,40] as a marker indicate the use of a mixture of oil and egg (*tempera grassa*) for pigment application.

Py-GC/HRMS analyses of layers (d), (e), and (f) reveal the presence of fatty acids, including dicarboxylic acids indicative of a siccative oil and protein compounds. The high percentage of pyrroles and the presence of diketopyrrole, derived from the thermal degradation of hydroxyproline, identify the use of animal glue, according to the scientific literature [37,40]. Small percentages of abietanes (dehydroabietic acid, 7-methoxy tetrahydroabietic acid, and 7-oxo-dehydroabietic acid) are also present in the pyrogram and are attributed to a terpenic resin. The presence of the resin in the lower part of the sample suggests that impregnation of the wood support may have been performed. In addition, polysaccharide compounds (guaiacyl- and syringyl-type phenols and derivative compounds) were detected, originating from the thermal degradation of lignin in the wood substrate [41].

#### 3.2.3. EH13 Sample

**Figure 13:**
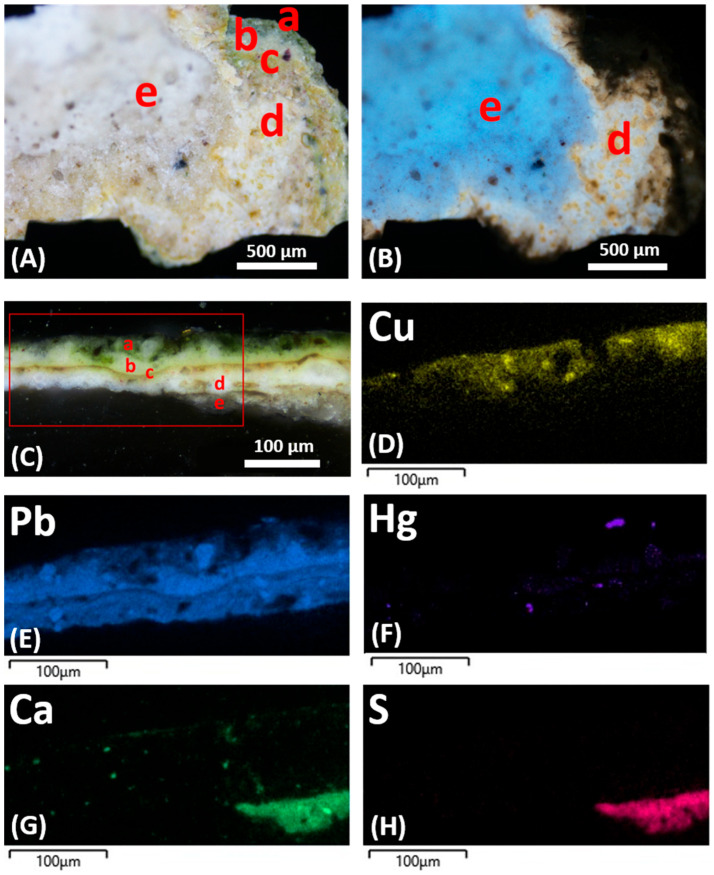
EH13 sample: (**A**) fragment back under VIS; (**B**) fragment back under UV; (**C**) stratigraphy on polished cross-section; (**D**–**H**) SEM-EDS distribution maps.

The cross-section of the sample reveals a sequence consisting of five layers, starting from the surface as follows (Figure 13):

(a) A green-colored layer, around 30 μm thick.
(b) A white, fine, and uniform layer with a thickness of around 20 μm, containing sporadic and very fine red particles.
(c) A brown-orange layer, very thin (around 5 μm), made of clay particles. The observation of some Au particles on the sample as it is, but lost in cross-section preparation, suggests that this layer is a preparation for the application of gilding.
(d) A white-colored layer, around 30 μm thick, containing sporadic and minute red particles with dimensions of a few micrometers.
(e) A 40 μm thick layer, white under visible light and showing blue UV fluorescence, suggesting that this layer corresponds to a preparation layer applied prior to the pictorial finish.

The elemental composition determined by SEM-EDS analyses (Figure 13) shows that the green layer (a) mainly contains Cu. Pb, relating to white lead, was also detected in this layer and was prevailing in layers (b) and (d). In these layers, secondary red pigments are testified by the presence of traces of Hg, denoting cinnabar, and Al, Si, K, and Fe coming from ochres. The latter elements were detected in the (c) layer, confirming that this was a red bolus used for as preparation under gold leaves. As in the case of EH12, a ground layer (e), made of Ca and S, suggests an original gypsum-based preparation layer applied to the wood substrate.

**Figure 14:**
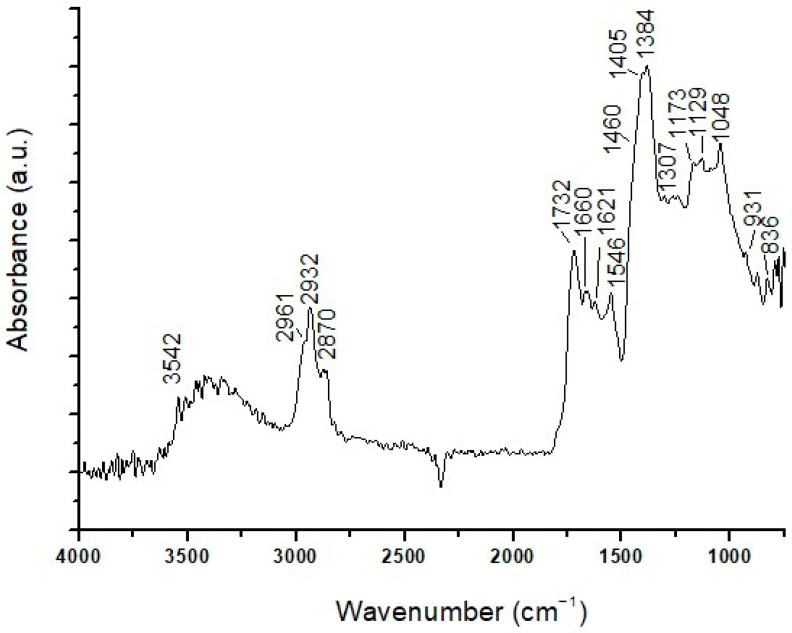
ATR-FTIR spectrum of the EH 13 sample.

ATR-FTIR analyses did not differentiate the composition of the individual layers of the sample due to their very small thicknesses. Overall, the acquired ATR-FTIR spectra (Figure 14) indicate the presence of infrared peaks at 3542, 1405, 1173, and 1048 cm^−1^ related to lead carbonate, and at 1129, 1048, and 931 cm^−1^ attributed to the presence of aluminosilicates as components of an earth pigment. Peaks at 2961, 2932, 2870, and 1732 cm^−1^ denote a lipidic component. Spectroscopic analyses also detected traces of proteinaceous material through weak peaks at 3400, 2932, 2870, 1660, 1546, and 1460 cm^−1^; traces of calcium oxalates, corresponding to peaks at 1621 and 1307 cm^−1^ [42]; and carboxylates, with a peak at 1534 cm^−1^. The traces of protein compounds and calcium oxalates probably relate to the gypsum-based ground layer as a preparation applied to the wood prior to the paint finish, accounting for the blue UV fluorescence observed for this layer. Finally, the presence of malachite was detected through an intense peak at 1384 cm^−1^ and a weak signal at 836 cm^−1^, according to the green color of the (a) layer and the presence of Cu detected by SEM-EDS.

PY-GC/HRMS analysis of sample portions including layers (a) and (b) shows the presence of mono- and dicarboxylic fatty acids. The high proportion of ΣD (mainly azelaic, suberic, and sebacic acids), the A/P ratio >1, and the high amount of azelaic acid indicate the use of a siccative oil (probably walnut oil) as the binder.

Analysis of the overall layers (c), (d), and (e) detected the presence of mono- and dicarboxylic acids and protein compounds. According to the literature [36,40,43], the high content of dicarboxylic acids, an A/P ratio >1, and an azelaic acid amount >10% indicate the use of a siccative oil as a binder, while the presence of pyrroles and a high percentage of diketopyrrole indicate the presence of animal glue. The latter further supports that the ground layer (e) is a preparatory layer. Traces of lignin degradation compounds are present in the pyrogram and they likely come from the wooden substrate.

### 3.3. Ecce Homo 2 Statue

#### EH5 Sample

**Figure 15:**
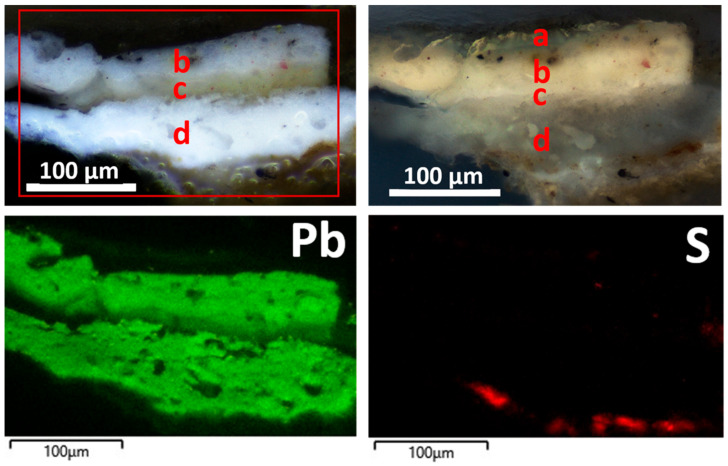
EH5 sample: stratigraphic sequence under VIS and UV light (**top left** and **right**, respectively); distribution maps of Pb and S detected in the red-outlined area, indicative of white lead in the painted layers and gypsum in the ground preparation layer (**bottom**).

Sample cross-section under PLM shows the following layers (Figure 15):

(a) A surface varnish layer, about 15 μm thick, visible only discontinuously under UV light.
(b) A layer about 40 μm thick, white in visible light and yellowish under UV light, with a fine and uniform structure and diffusely containing minute red particles of a few micrometers.
(c) A thin yellow varnish layer (about 20 μm), appearing white under UV light.
(d) A layer over the wood, around 90 μm thick, appearing white, finely and uniformly grained, and containing very sporadic and small red particles; under UV light, it shows a yellow-grayish fluorescence.

A further layer under layer (d) was highlighted by SEM-EDS and made of Ca and S, corresponding to gypsum probably used as a preparation layer. White lead was suggested in layers (b) and (d), made of mainly Pb along with traces of Si, Al, and Fe, which account for the presence of ochre pigments.

The µATR-FTIR analyses of the outer layers, including layers (a), (b), and (c), identified a resin with characteristic peaks at 2937, 2861, 1704, 1419, 1322, 1157, 1103, 1034, and 934 cm^−1^, and lead carbonate through infrared bands at 3386, 2932, 2861, 1419, 1322, 1160, 1110, 1038, 934, and 849 cm^−1^. Intense infrared peaks are also evident at 1121, 1060, and 931 cm^−1^, corresponding to earth aluminosilicates used as red pigments.

In the lower layers (d) and (e), infrared bands typical of lead carbonate are evident. Lipid compounds with bands at 2935, 2865, and 1728 cm^−1^ and carboxylates with a peak at 1530 cm^−1^ are also present. Finally, bands at 1643, 1560, and 1460 cm^−1^ were detected, indicating traces of protein compounds, probably responsible for the blue fluorescence observed for this layer under UV light. The presence of proteinaceous material probably relates to the gypsum-based layer (e) as a preparation layer for receiving the pictorial finish. The pyrogram of the sample shows the presence of several natural compounds. Dehydroabietic acid and 7-oxo-dehydroabietic acid indicate the presence of aged and oxidized colophony, while the presence of retene indicates that the terpenic resin was subjected to heating. Mono- and dicarboxylic acids were also detected, and the A/P ratio together with the percentage content of azelaic acid suggests the use of a siccative oil as the pictorial binder. A considerable pyrrole content and a high percentage of diketopyrrole indicate the use of animal glue, likely in the preparatory layer. Finally, a considerable percentage of polysaccharide compounds was detected. Guaiacyl- and syringyl-type phenols and derivative compounds from the thermal degradation of lignin can be attributed to the wooden substrate. However, due to the large amount of polysaccharide derivatives and the presence of markers such as 1,2,4-trimethoxybenzene and permethylated saccharidic acids, it is also possible to hypothesize the use of a plant gum [44,45], which is usually difficult to identify by Py-GC/HRMS analysis due to its structure, especially in complex samples and in the presence of other polysaccharide sources.

## 4. Discussion

The results obtained for the investigated samples are summarized in Table 3, Table 4 and Table 5.

Microscopic observations show that the stratigraphies of the investigated samples include multiple pictorial and varnish layers, highlighting various episodes of intervention on the artifacts.

The main inorganic pigment identified by optical microscopy, SEM-EDS, and FTIR in all the flesh tone layers of the three statues is white lead, very commonly used in the artistic field until the 19th century [46]. Only in one case (SO4 sample, layer (c)) was barium white found; it is known to have been used starting from 1782 and introduced into commerce between 1810 and 1820 [47].

White pigments are mixed with red ochre particles, often black carbon, and sometimes cinnabar. These additional pigments are randomly distributed within the sequence layers and are therefore not significant for discriminating original and subsequent paintings. They were found in different proportions or grain sizes, likely related to the different flesh tones imparted to the statues in renewed polychromies during various remakes. Malachite was recognized in the decoration pattern with green and red bands of the loincloth of the Ecce Homo 1 statue (EH13 sample), which stratigraphy revealed to be non-original. A previous remake of the loincloth probably consisted of gold leaf, whose sporadic relicts were observed on the samples as collected but were lost during the cross-section preparation. The elemental composition of the orange-colored layer (c) in this sample, exclusively made of Al, Si, K, and Fe, is ascribable to aluminosilicates and accounts for a red bolus layer applied to receive the gold leaf finishing. Finally, a mixture of bone black, ochre, and black carbon was identified in the repainted layer (c) of the EH12 sample, corresponding to the hair.

ATR-FTIR spectrophotometric analyses identified the main organic compounds in the samples, whose presence was denoted under UV light and, wherever possible, enabled determination of their stratigraphic distribution thanks to the use of an IR microscope. They consist of lipids and proteinaceous compounds relating to binders, while terpenic resins were detected as varnishes. Oxalates were found only in one sample (EH13), having a possible origin from protein binders. Spectrometric results obtained by Py-GC/HRMS analyses, based on the detection of the main markers in the pyrograms of the samples, their semi-quantitative evaluation, and their specific ratios, show that siccative oil was almost exclusively used as a binder, both in layers considered original and in those evidently repainted. In some cases, namely the EH11 and EH13 samples from Ecce Homo 1, Py-GC/HRMS technique even made it possible to hypothesize that the drying oil used was linseed oil and walnut oil, respectively.

Only in two samples from S. Onofrio and Ecce Homo 1 were oil and egg found in the repainted layers (SO4 and EH12), suggesting the use of a *tempera grassa* technique. Varnish layers in all samples consist of colophony, which in most samples appears to have been subjected to heating before application and affected by oxidation processes due to aging over time (except for the SO1 sample).

As regards the original layers, the following indications can be drawn from the analysis of the overall results.

A ground layer was identified in samples from both Ecce Homo statues, which was probably applied as a preparation of the support for the application of the pictorial films.

In the case of the Ecce Homo 1 statue, a ground layer made of Ca and S was detected in the EH12 and EH13 samples, namely layers (f) and (e), respectively, suggesting a gypsum-based preparation. It was also found to contain siccative oil and animal glue, as detected by PY-GC/HRMS. Layer (d), in direct contact with this ground layer in the EH13 sample, could be the original one, made of lead carbonate with very sporadic small red particles and a siccative oil binder.

Original painted layers seem to be absent in the EH11 and EH12 samples. In the former, no preparation layer was observed; therefore, it was probably lost, suggesting that the bottom layer (c), in direct contact with the wood substrate, is a remake. In the case of the EH12 sample, it should be noted that the microstructure of layer (e), just above the preparation layer, appears to be very different from that of layer (d), which is in direct contact with the wood in the EH13 sample. On the contrary, it is similar to that observed for the repainted layer (b) at the top of the sequence in the EH11 sample. Therefore, a correlation between these two layers can be established, implying that layer (e) of the EH12 sample, although directly on the ground preparation layer, is also a remake. On the contrary, layer (d) in the EH13 sample is probably the original painting.

The complex of stratigraphic sequences observed in the analyzed samples highlights three repainting episodes of the Ecce Homo 1 statue, where the black layer (c) under the last flesh remake ((b) layer) in the EH12 sample probably comes from color smearing of hair in the area adjacent to the flesh sampling point.

The Ecce Homo 2 statue also exhibits a ground layer (e) with a composition similar to that found for Ecce Homo 1. In this case, the pictorial layer(d) and varnish layer (c) could be original, while the superimposed layers (b) and (a) are clearly repainted.

In the case of S. Onofrio, layer (c) just above the wood in the SO4 sample contains barium white, which indicates a repainting dating to after the end of the 18th century or the early decades of the 19th century, when this pigment began to be used by artists. It goes without saying that the overlying pictorial layer (a) and varnish (b) also come from a remake. Moreover, layers (a) and (c) differ in their binders, namely siccative oil and *tempera grassa*, respectively. No preparation layer was observed. As regards layer (d), in direct contact with the wood in the SO1 sample, it could be original; in such a case, it would mean that no preparation layer was used. On the other hand, the microscopic features and UV fluorescence color, quite similar to those of the (a) layer in the SO4 sample, suggest that this paint layer in the SO1 sample is also a remake and that the original finish is absent.

On the basis of the analytical evidence from both S. Onofrio samples, we can deduce that the statue underwent at least three repainting episodes. We have no unambiguous elements to support whether a preparation of the wood substrate was applied and then lost with the original pictorial layers, or whether the painting was applied directly on the wood.

The treatment of wood supports in polychrome artworks is a practice implemented since ancient times. A preparatory layer made of calcium carbonate, along with animal glue as a medium to bind calcite particles, has been found on various types of artifacts from ancient Egypt, such as coffins [2,48], naos [3], and statuettes [6,22,49]. It had the function of making the surface uniform and smooth prior to application of painting and gilding layers by filling any wood defects or irregularities [50], as well as improving adhesion and protecting the brittle paint layers from contraction and swelling of the wooden support [51]. Cennino Cennini [52], between the 14th and the 15th centuries, precisely describes how to make a preparation based on gypsum and animal glue, recommending its application as a fundamental preparatory base for painting on wood.

Romanesque polychrome wood sculptures in Italy exhibit preparation layers made of gypsum mixed with animal glue, and collections from Palazzo Venezia in Rome, which range from the 12th to the 18th century and cover many countries, such as Italy, Spain, Germany, and the Netherlands, show gypsum used in sculptures south of the Alps and either gypsum or calcium carbonate in those from northern Europe [53]. Gypsum and calcite seem to be the main compounds used in ground layers in most samples from statues of Extremadura cultural heritage (Spain), dating from the 13th to the 18th centuries [10]. Sometimes, lead white has been found as a primer under a calcium carbonate ground layer in the latter or in preparatory layers of the Baroque sculptures of Gaetano Patalano in Southern Italy, which were selectively based on lead white under the skin and hair and on gypsum elsewhere [12].

Indeed, ground preparatory layers on polychromatic wood are documented through the centuries on various types of artifacts, from statuary dating from the Middle Ages to the 19th century [5,7,9,23,54] to Portugues “talha dourada” dating between the 17th and 18th centuries [55].

## 5. Conclusions

The analytical study of some examples of the Baroque polychromy on wood from Southern Italy was conducted, in order to identify and characterize ground layers, paint layers, and binding media. Microscopic, spectroscopic, and spectrometric techniques were successfully implemented within an integrated methodology capable of acquiring accurate and exhaustive analytical knowledge, with an extremely limited impact on the analyses, thanks to the use of advanced techniques with minimal destructiveness, complying with the fundamental requirement of preserving the integrity of works of art.

In particular, using the same sample cross-sections, optical microscopy under visible and UV light and low-pressure SEM allowed the identification of stratigraphic sequences and morphological–structural features of the pictorial layers, along with optically detectable components. Chemical composition and elemental distribution determined through SEM-EDS microanalyses and FTIR analysis of inorganic components led to the identification of mineral pigments within each layer. In addition, FTIR spectrophotometry and microscopy in ATR mode were used to detect the main organic compounds in the samples and, wherever possible, their stratigraphic distribution. Finally, Py-GC/MS high-resolution analyses made possible the precise identification of the nature of organic compounds through qualitative characterization of the main markers present in the pyrograms of the samples, their semi-quantitative evaluation, and the calculation of specific ratios, while needing only minute amounts of sample powder.

The results obtained from these combined techniques highlighted that two of the investigated statues (Ecce Homo 1 and 2) had a ground layer likely functioning as preparation of the wood substrate prior to the application of the pictorial finish. This layer was made of gypsum and animal glue. Original layers in direct contact with these preparation layers were identified and were found to have siccative oil as a binder. Repainting layers were also found to contain siccative oil binder markers, while in some cases they revealed a mixture of oil and egg, denoting a pictorial technique based on *tempera grassa*. As regards pigments, flesh tone layers, which were mainly investigated, were found to be made of white lead as the main pigment, with additions of red ochre and sometimes cinnabar, in both original and overpainted layers. Red pigments were often mixed with carbon black, and various tones resulted from different pigment proportions. White barium, malachite, and bone black were also identified in some repainted layers. Therefore, because pictorial materials were almost the same across pictorial sequences, differentiation between original and overpainted layers was fundamentally supported by stratigraphic features. Only in the case of the S. Onofrio statue was the presence of barium white in a sample crucial for excluding that the originality of the layer in direct contact with the wood substrate. For this sculpture, microscopic and stratigraphic features were not unequivocal in ascertaining the presence of original layers on the statue, and no preparation layer was observed.

Finally, the multi-analytical study was effective in gaining important knowledge of the materials and techniques of the painted wood sculpture of the Baroque period in Southern Italy, which sheds better light on this artistic production, poorly studied so far, and more generally on the polychromy applied to wood. The study also provided evidence of layers repainted over time, which is fundamental in driving conservation processes.

## Figures and Tables

**Table 1:** Main compounds and retention time (RT) identified in the Py-GC/HRMS chromatograms of the samples. DME: dimethyl ester; ME: methyl ester.

RT	Compound	SO1	SO1	SO4	SO4	EH11	EH12	EH12	EH13	EH13	EH5
		(Overall)	(b–d)	(a, b)	(b, c)	(Overall)	(a–c)	(d–f)	(a, b)	(c–e)	(Overall)
3.73	1H-pyrrole, 1-methyl							V		V	V
6.19	Phenol										V
6.22	Heptanoic acid, ME	V		V	V	V	V	V			
7.19	Pyrrole, 1,2,5-trimethyl										V
7.30	Guaiacol				V			V			V
8.01	Octanoic acid, ME	V			V	V					
8.03	Indole							V			
8.92	*p*-methylguaiacol							V			V
9.76	*p*-ethylguaiacol							V			V
10.30	Permethylated saccharidic acid										V
10.66	*p*-vinylguaiacol									V	V
10.71	Decanoic acid, ME			V		V			V	V	V
10.98	Heptanedioic acid, DME	V	V	V	V	V		V			V
11.25	Syringol				V						V
12.27	Octanedioic acid, DME	V	V	V		V	V		V	V	V
12.35	1,2,4-trimethoxybenzene										V
13.37	Nonanedioic acid, DME	V	V	V	V	V	V	V	V	V	V
	(azelaic acid, DME)										
13.68	4-vinylsyringol							V			V
14.12	Methoxyeugenol							V			V
14.55	Decanedioic acid, DME	V	V	V	V	V				V	V
15.13	Diketopyrrole							V		V	V
15.32	Tetradecanoic acid, ME						V				V
15.58	Undecanoic acid, ME	V	V	V	V	V		V	V	V	V
16.01	Hexadecanitrile			V			V				
16.33	Pentadecanoic acid, ME				V	V		V	V	V	V
17.02	9-hexadecenoic acid, ME			V							
17.21	Hexadecanoic acid, ME	V	V	V	V	V	V	V	V	V	V
	(palmitic acid, ME)										
18.21	Heptadecanoic acid, ME	V	V	V	V	V		V	V		V
18.87	Oleic acid, ME	V	V	V		V	V	V	V	V	V
19.11	Octadecanoic acid, ME	V	V	V	V	V	V	V	V	V	V
	(stearic acid, ME)										
19.92	Retene			V	V	V	V	V			V
20.93	Dehydroabietic acid, ME	V		V	V	V	V	V			V
21.82	7-methoxy-tetradehydroabietic					V	V	V			
	acid, ME										
22.73	7-Oxodehydroabietic acid, ME	V		V	V	V	V	V			V
24.03	Cholesterol, ME			V							
24.67	Cholest-3,5-diene			V							
24.89	Cholesta-3,5-dien-7-one			V							

V indicates the presence of the specific compound in the sample.

**Table 2:** Molecular markers suggested in the literature and identified in samples by PY-GC/HRMS analysis for the identification of binding media, their percentages, and ratios. FA: fatty acids; ΣD: dicarboxylic acids; A: azelaic acid; P: palmitic acid; S: stearic acid.

Compound	SO1 (Overall)	SO1 (b–d)	SO4 (a, b)	SO4 (b, c)	EH11 (Overall)	EH12 (a–c)	EH12 (d–f)	EH13 (a, b)	EH13 (c–e)	EH5 (Overall)
FA	96.98	92.55	73.65	70.68	98.01	48.38	30.69	97.90	80.79	66.50
ΣD	56.51	73.49	39.65	58.72	42.64	16.15	6.53	56.38	45.08	35.83
Proteins	/	/	6.89	/	/	5.95	6.09	/	19.01	10.67
Polysaccharides	/	/	/	7.58	/	/	19.80	/	0.20	38.71
Abietanes	2.66	/	1.43	1.25	1.59	22.74	1.62	/	/	2.95
A	40.80	43.75	1.31	34.92	30.94	1.52	3.07	46.58	11.43	12.88
P/S	4.05	6.69	1.11	2.13	1.27	2.05	1.89	2.96	3.18	1.26
A/P	1.53	2.00	0.10	2.74	1.28	0.07	0.39	1.55	0.26	1.05

**Table 3:** Summary of the results obtained for S. Onofrio.

Sample	PLM	SEM-EDS	ATR-FTIR	Py-GC/HRMS	Pigments	Layers’ Attribution
SO1	(a) discontinuous varnish		resin	colophony		Not original
	(b) homogeneous white pictorial layer, from 15 up to 20 μm thick, with sporadic red particles sized between 2 and 20 μm	Pb, Si, Al, Mg, Ca, K, Fe, and Ti	Layers b–d: lead carbonate, lead carboxylates, lipids, and aluminosilicates	layers b–d: siccative oil	white lead and ochre	Not original
	(c) very thin, discontinuous dirt deposition layer	Ca, Al, Si, K, Mg, Fe, and Na			earthy deposit	Not original
	(d) 20 μm thick layer, white in VIS light, with straw-yellow UV fluorescence, containing very few fine red particles with sizes from some microns up to 15–20 μm	Pb, Si, Al, Mg, Ca, K, Fe, and Ti			white lead and ochre	Original
SO4	(a) fine compact white pictorial layer, from about 25 to 55 μm thick, yellowish under UV, containing diffuse red particles with sizes up to about 20 μm	Pb, Si, Ca, Al, K, Fe, and Hg	Layers a–c: resin, lead carbonate, lipids, lead carboxylates, and red ochre	oil and egg (*tempera grassa*)	white lead and red ochre	Not original Repainting with *tempera grassa*
	(b) brown varnish, from about 8 to 15 μm thick			colophony		Not original
	(c) pictorial layer, from 10 to about 60 μm, white-yellowish at the bottom and yellowish at the top, with a fine matrix and sharp-edged coarse crystals	Ba, S, Pb, Si, Ca, Al, K, and Fe		siccative oil	barium white, white lead, and red ochre	Not original Repainting with barite

**Table 4:** Summary of the results obtained for Ecce Homo 1.

Sample	OM	SEM-EDS	ATR-FTIR	Py-GC/HRMS	Pigments	Layers’ Attribution
EH11	(a) varnish		Layers a–c: resin, lipids, lead carbonate, and lead carboxylates	Layers a–c: colophony and siccative oil (linseed oil)		Not original
	(b) white pictorial layer, yellowish under UV, 110 μm thick, with a fine matrix containing a few sharp-edged translucent crystals, red and coarse black particles	Pb, Si, Al, Mg, Fe, and Hg			white lead, black carbon, ochre, and cinnabar	Not original
	(b1) very thin discontinuous varnish					Not original
	(c) white pictorial layer, yellowish under UV, 150 μm thick, with a fine matrix containing red and fine black particles	Pb, Si, Al, Mg, Fe, and Hg			white lead, ochre, cinnabar, and black carbon	Not original
EH12	(a) discontinuous varnish layer			colophony		Not original
	(b) white pictorial layer, 90 μm thick, with diffuse minute red particles and sporadic smaller black particles	Pb, Hg, and Al	Layers b–c: lead carbonate, aluminosilicates, lipids, and lead carboxylates	Layers b–c: siccative oil and egg (*tempera grassa*)	white lead, cinnabar, ochre, and black carbon	Not original Repainting with *tempera grassa*
	(c) 20 μm thick layer, with very intense black pigment and diffuse carbonaceous particles	Ca, P, Al, Si, Mg, and Fe			black bone, ochre, and black carbon	Not original
	(d) white layer, 75 μm thick, with orange-red particles and translucent sharp-edged coarse crystals	Pb and Al	Layers d–f: lead carbonate, lipids, aluminosilicates, and proteins	Layers d–f: siccative oil, animal glue, and resin	white lead and ochre	Not original
	(e) fine white pigment layer, about 70 μm thick, including sharp-edged translucent coarse crystals, sporadic fine yellow–red particles and diffuse coarser black particles	Pb, Al, Si, Mg, and Fe			white lead, ochre, and black carbon	Not original, being similar as EH11 (b) layer
	(f) very thin white layer, <10 μm thick, with bluish fluorescence	Ca and S			gypsum (preparation)	Original
EH13	(a) green pictorial layer, 30 μm thick	Cu and Pb	Layers a–e: lead carbonate, aluminosilicates, lipids, proteins, calcium oxalate, carboxylates, and malachite	Layers a–b: siccative oil (walnut)	white lead and malachite	Not original
	(b) white fine pictorial layer, 20 μm thick, with sporadic very fine red particles	Pb, Hg, Al, Si, K, and Fe			white lead, ochre, and cinnabar	Not original
	(c) very thin brown-orange layer, 5 μm thick, with sporadic Au particles on the sample as it was, but lost during cross-section preparation	Al, Si, K, and Fe		Layers c–e: siccative oil, animal glue, and lignin degradation compounds.	red bolus	Not original
	(d) white layer, 30 μm thick, with sporadic fine red particles	Pb, Hg, Al, Si, K, and Fe			white lead, cinnabar, and ochre	Original
	(e) white layer, 40 μm thick, with UV blue fluorescence	Ca and S			gypsum (preparation)	Original

**Table 5:** Summary of the results obtained for Ecce Homo 2.

Sample	PLM	SEM-EDS	ATR-FTIR	Py-GC/HRMS	Pigments	Layers’ Attribution
EH5	(a) discontinuous varnish layer, about 15 μm thick		Layers a–c: resin, lead carbonate, and aluminosilicates	Layers a–e: colophony, siccative oil, animal glue, lignin degradation compounds, and plant gum		Not original
	(b) white layer, about 40 μm thick, containing red particles	Pb, Si, Al, and Fe			white lead and ochre	Not original
	(c) very thin brown varnish, about 20 μm thick					Original
	(d) discontinuous white layer, about 90 μm thick, containing very sporadic small red particles	Pb, Si, Al, and Fe	Layers d–e: lead carbonate, lead carboxylates, lipids, and proteins		white lead and ochre	Original
	(e) thin discontinuous layer evident only by SEM-EDS	Ca and S			gypsum (preparation)	Original

## Data Availability

The original contributions presented in the study are included in the article, further inquiries can be directed to the corresponding author.
